# Returning for HIV Test Results: A Systematic Review of Barriers and Facilitators

**DOI:** 10.1155/2016/6304820

**Published:** 2016-12-15

**Authors:** Patrice Ngangue, Emmanuelle Bedard, Hervé Tchala Vignon Zomahoun, Julie Payne-Gagnon, Claudia Fournier, Jeannette Afounde, Marie-Pierre Gagnon

**Affiliations:** ^1^Faculté des Sciences Infirmières, Université Laval, 1050 avenue de la Médecine, Pavillon Vandry, Ville de Québec, QC, Canada G1V 0A6; ^2^Population Health and Optimal Health Practices, CHU de Québec Research Center, 10 rue de l'Espinay, D6, Quebec City, QC, Canada G1L 3L5; ^3^Université du Québec à Rimouski (UQAR), 1595 boul. Alphonse-Desjardins, UQAR, Campus de Lévis, Lévis, QC, Canada G6V 0A6; ^4^Quebec SPOR-SUPPORT Unit, 10 rue de l'Espinay, D6, Quebec City, QC, Canada G1L 3L5; ^5^Department of Family Health, Ministry of Public Health, Yaoundé, Cameroon

## Abstract

This systematic review aims to identify factors that facilitate or hinder the return for HIV test results. Four electronic databases were searched. Two independent reviewers selected eligible publications based on inclusion/exclusion criteria. Quantitative studies published since 1985 were included. Thirty-six studies were included in the final review. Individual level barriers included sociodemographic characteristics, such as being a male, of young age and low education level, risk behaviours such as injecting drugs, having multiple sexual partners, and psychosocial factors. Older age, higher education level, being a woman, having high self-esteem, having coping skills, and holding insurance coverage were identified as facilitators. Interpersonal barriers and facilitators were linked to risk behaviours of sexual partners. Contextual barriers included essentially the HIV testing center and its characteristics. This review identified the most important factors that need to be addressed to ensure that people return for their HIV test results.

## 1. Introduction

HIV testing and counselling (HTC) is the cornerstone of treatment, care, and prevention [1, 2]. It is particularly through HTC that the ambitious goal of 90, 90, 90 (90% of all people living with HIV will know their HIV status; 90% of all people with diagnosed HIV infection will receive sustained antiretroviral therapy; 90% of all people receiving antiretroviral therapy will have viral suppression), by 2020 [3] can be reached. Previously, most efforts were focused on voluntary counselling and testing (VCT) as the primary means of providing testing and encouraging people to become aware of their HIV status [2]. However, coverage remains low and many infected persons in both developed and developing countries remain undiagnosed. Despite the availability of rapid test with the possibility to have the results approximately in 20 to 30 minutes, in some contexts particularly, there are many who get tested but fail to return for their results [4–6]. For example, in the USA, data from HIV testing performed at publicly funded counselling and testing sites using conventional HIV enzyme immunoassay (EIA) testing from 1999 through 2002 found that 19% to 22% of people with positive preliminary HIV tests did not return for their test results [6]. In 2009, a survey conducted in 12 Sub-Saharan Africa countries with high HIV prevalence showed that only 10% of women and 12% of men were tested and received their test results [7]. In an evaluation of five years of routine program data in Vietnam, Hong et al. found a failure to return (FTR) rate of 3.5% [8]. In a study of female sex workers in China, Xu et al. found a FTR rate of 47.1% [9]. More recently, in France, Laanani et al. (2015) and Pahlavan et al. (2015), respectively, found a FTR rate of 6.5% in a study conducted in a free and anonymous screening center [10] and 14.5% in an HIV-positive population [11].

Identifying and targeting these people may improve the return rate for VCT and the proportion of individuals who are aware of their status. Therefore, the objective of this systematic review is to identify the factors that prevent people who are tested for HIV from returning for their results or facilitate their doing so.

## 2. Methods

This systematic review examines barriers and facilitators associated with returning for HIV test results in various types of populations and settings. The outcome variable, returning for HIV testing results, is dichotomous. From this point of view, some studies concerned factors associated with returning for HIV test results, while others focused on failure to return (FTR). This review was conducted in accordance with Preferred Reporting Items for Systematic Reviews and Meta-Analyses (PRISMA) statement guidelines [44].

### 2.1. Search Strategy

A comprehensive search strategy was developed to identify studies published between January 1985 (introduction of HIV tests) and June 2015. Four electronic databases were searched (PubMed/Biomed Central/Medline, Embase, PsycINFO, and Web of Science), combining terms related to HIV, counselling/testing, and return/failure to return. Retrieved references were imported into Endnote X7, and then duplicates were removed. The detailed search strategy is available upon request.

### 2.2. Study Selection

All identified records (*n* = 3,019) were initially screened by two independent investigators and verified by a third researcher. Eligible studies had to meet the following criteria: (1) be an original research study; (2) be written in English or in French; (3) report adolescents, adults, pregnant women, men who have sex with men (MSM), injecting drug users (IDUs), or female sex workers (FSWs); (4) include participants undergoing HIV tests; (5) use a quantitative method to assess return or failure to return for HIV test results; and (6) report a statistical association between a potential predictor/correlate and return or FTR. Study designs of interest were cross-sectional and longitudinal. No geographical restrictions were applied. The reference lists of the relevant articles were also reviewed for additional publications.

**Figure 1:**
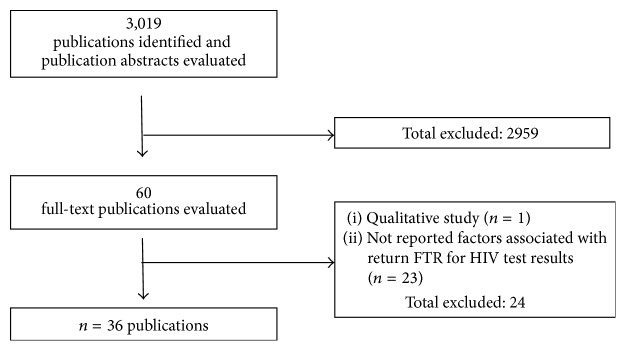
Flow chart of selected studies.

A short list of records was prepared and the full text reviewed independently by two authors. Citations that were clearly irrelevant were excluded. Uncertainties and disagreements about inclusion were resolved through discussion involving both investigators (see Figure 1 for flowchart of systematic review).

### 2.3. Data Extraction

Two authors independently extracted data from each study that fulfilled the inclusion criteria using a standard form. Study characteristics (name of the first author, year of publication, country in which the study was conducted, study design, sampling approach, participating characteristics, and HIV testing procedure) as well as key findings related to factors associated with return or failure to return for HIV test results were extracted. Any factors analyzed associated with FTR or return for HIV test results were listed, and the results of multivariate statistical tests for association (odds ratio) were noted. For studies where a multivariate statistical test was not done, the results of bivariate analyses were noted. When the result of the measure of association in multivariate analysis was not significant and not reported by authors, the factor was not considered in the synthesis.

### 2.4. Quality Assessment

The Newcastle-Ottawa Scale (NOS) for cohort studies and an adapted form of the Newcastle-Ottawa cohort scale for cross-sectional studies were used to assess methodological quality. NOS is a tool for assessing the quality of nonrandomized studies to be used in a systematic review [45]. Each study is judged with a “star system” on three points: the selection of study groups, the comparability of the groups, and the ascertaining of exposure or outcome. Studies for which at least five out of nine items on the NOS were deemed satisfactory and in which appropriate statistical analysis (e.g., multivariate controlling for confounders) was conducted were considered to be of sufficient methodological quality and included in the review (maximum score of 10 for cross-sectional studies and 9 for cohort studies). At each stage of the quality assessment, the reviewers discussed among themselves until a consensus was reached on which studies to include.

### 2.5. Data Synthesis and Combined Effect Sizes Associated with Return

Factors associated with either return or failure to return may be arranged into barriers and facilitators inspired by the Socioecological Model (SEM), which is a framework to examine the multiple effects and interrelatedness of environmental, contextual, and social factors on individual behaviour [46, 47]. Recognizing that most public health challenges are too complex to be adequately understood and addressed from single level analyses, the SEM includes a more comprehensive approach that integrates multiple levels of influence to impact health behaviour and ultimately health outcomes. These levels of influence include intrapersonal and interpersonal factors, organizational factors, and structural factors. In this review, due to their small number, organizational and structural factors were grouped as contextual factors.

Since we anticipate a potential variability of the methodology (e.g., measures of studied factors) across the included studies, we used a random-effects model based on the inverse variance method to estimate the pooled odds ratio (OR) for each factor potentially associated with returning for HIV test results and its 95% confidence interval (CI) [48, 49]. The Higgins's *I* ^2^ statistic was used to quantify the percentage of the variability in individual effect size estimates which is attributable to the heterogeneity [50, 51]. This heterogeneity was tested using a chi-squared test [50, 51]. Moreover, we performed sensitivity analysis by removing the included studies from the pooled size estimation one at a time. These analyses allowed us to explore the individual contribution of each study to the heterogeneity in the meta-analysis. When we could not explain the heterogeneity, we have interpreted the pooled effect size estimates with caution because these effect sizes would be explained by other factors, which were not taken into account in our analyses. A *p* value of less than 0.05 was considered statistically significant. Analyses were performed in Review Manager (version 5.3).

## 3. Results

### 3.1. Study Selection

The primary search strategy identified 3,019 potentially relevant citations. After the removal of duplicates and the initial title and abstracts screening, 60 citations were kept for the full-text review. Studies were excluded if they did not report quantitative results (*n* = 1) or just reported the rate of return or failure to return (FTR) without assessment of associated factors (*n* = 23). The remaining 36 studies were appraised for their methodological quality and included in the analysis. No study was excluded on the basis of quality assessment. A flow chart illustrating the selection process is shown in Figure 1.

### 3.2. Study Characteristics

Table 1 provides a brief overview of the key characteristics of the included studies. Of the 36 included studies, 10 were longitudinal cohort studies and 26 were cross-sectional studies. Seven of the studies were carried out in Sub-Saharan Africa; seventeen in the USA; three in Australia; six in Asia; and one in Brazil. Populations under study were diverse, including general population (*n* = 16), pregnant women (*n* = 5), injecting drug users (*n* = 3), men who have sex with men (*n* = 3), high-risk heterosexual individuals (*n* = 2), HIV-positive individuals (*n* = 2), factory workers (*n* = 2), individuals with psychiatric problems (*n* = 2), adolescents (*n* = 1), HIV-negative individuals (*n* = 1), and female sex workers (*n* = 1). The outcome of interest was dichotomous, with 20 studies focused on failure to return and 16 on return for HIV test results.

### 3.3. Quality Appraisal Results

Studies were generally of high quality (see Table 2). A total of 3 cohort studies scored 9/9, one study scored 8/9, and 6 studies scored 7/10. For the cross-sectional studies, one study scored 9/10, 11 studies scored 8/10, 4 studies scored 7/10, 4 studies scored 6/10, one study scored 5/10, and one study scored 4/10.

### 3.4. Barriers and Facilitators of Returning for HIV Test Results

In total, 236 factors associated with returning for HIV test results were identified. Among these, 123 factors were reported as barriers and 70 as facilitators. The association was not statistically significant for 72 factors. At the individual level, factors were classified into sociodemographic characteristics (*n* = 78), risk behaviours (*n* = 64), perceived risk (*n* = 9), HIV knowledge (*n* = 7), reasons for visit/testing (*n* = 11), HIV test results (*n* = 13), history of testing (*n* = 11), psychosocial factors (*n* = 5), and other individual factors (*n* = 4). Factors grouped at the interpersonal level were risk partner behaviours (*n* = 7), social support (*n* = 6), knowledge of person with HIV (*n* = 2), domestic violence (*n* = 3), and other interpersonal factors such as partner age (*n* = 1), years in couple (*n* = 1), and communication within the couple (*n* = 2). Contextual factors comprised the type of clinic attended (*n* = 6), year of testing (*n* = 1), and characteristics of the testing center, such as availability of counselling (*n* = 1), condom distribution (*n* = 1), clinic visit (*n* = 1), confidential testing (*n* = 1), and location of the testing center in the same city as treatment center (*n* = 1) (see Table 3).

### 3.5. Individual Level

#### 3.5.1. Sociodemographic Characteristics

Age was the most reported factor (*n* = 16). This factor has been reported as a barrier to returning for results in 7 studies [12, 19, 27, 28, 30, 36, 37] and as a facilitator in 5 studies [13, 22, 35, 36, 39]. The association between age and returning for HIV test results was insignificant in 10 studies [10, 12–14, 26, 28–30, 35, 41]. In these studies, being 30 years of age or over was reported as a facilitator in 4 studies [13, 22, 35, 36] and as a barrier in just a single study [27]. On the other hand, having less than 30 years of age was reported as barrier in 6 studies [12, 19, 27, 28, 30, 36] and as facilitator in 2 studies [22, 36].

Level of education was reported in nine studies. In 4 studies [15, 17, 26, 30], it was reported as a barrier to returning for HIV test results, especially for people with no education or a low level of education. In 3 studies [9, 34, 39], it was reported as a facilitator for those with a medium or high level of education.

In studies with a mixed population (women and men) when sexual orientation was reported (*n* = 4), being heterosexual or bisexual appeared as a barrier to returning for HIV test results [11, 36, 37].

Marital status was reported in six studies. In these studies, being married or living in a couple [8] and being a widower [8] emerged as facilitators of a return for results.

#### 3.5.2. Risk Behaviours

Several risk behaviours were positively or negatively associated with a return for results. The number of sexual partners during the last 6 to 12 months was reported in 8 studies. In 5 of these studies [8, 10, 26, 27, 37], having more than 5 sexual partners was reported as a barrier to returning for HIV test results. Otherwise, having a single sexual partner during the last 6 to 12 months [22, 37] was not significantly associated with a return for results. Using a condom has been reported as both a barrier [30] and as a facilitator [26, 41], but in most cases, the association was not significant [14, 37, 39]. Having a history of STIs was reported in nine studies. However, it was reported equally as a barrier [16, 19, 29, 41] or a facilitator [13, 22, 25, 31] as regards a return for HIV test results.

#### 3.5.3. Perceived Risk

Perceived risk has been reported in nine studies. In those studies, having low perceived risk [26] (*n* = 1) and not seeing oneself at risk [10, 27] (*n* = 2) was reported as a barrier to a returning for test results. However, this result is somewhat controversial because one of these two studies showed that having high-perceived risk [10] was a barrier to a return for results, and having a medium perceived risk was reported at the same time as a barrier in one study [14] and as a facilitator in another [33]. In addition, the association was insignificant for 4 studies [10, 35, 38, 39].

#### 3.5.4. Psychosocial Factors

The association between the return for HIV test results and psychosocial factors showed divergent results (*n* = 5). For instance, not believing in self-prevention from HIV [26], believing that HIV can be cured [29], and thinking that a medical follow-up can improve the course of HIV [29] were reported as barriers to a return for test results. However, having high self-esteem [33] and positive coping skills [33] appeared as a facilitator for a return for results. Feeling anxious about HIV was reported as a barrier [15, 42] to a return for HIV test results.

#### 3.5.5. Health Coverage

The association between the existence of health coverage and a return for results was studied in two articles. Having health coverage [24] appeared as a facilitator and not having health coverage as a barrier [31].

### 3.6. Interpersonal Level

Interpersonal factors were reported in 18 studies. The most common factors were risk behaviours of the sexual partner. The association of these factors with a return for results was investigated in seven publications. The HIV status of the sexual partner [8, 19, 41] (*n* = 3) or being a client of a sex worker [8] (*n* = 1) was identified as a facilitator for a return for HIV test results in three studies. Having a sexual partner who is a sex worker [8, 41] (*n* = 2), having a partner who drinks alcohol [28] (*n* = 1) or consumes drugs [41] (*n* = 1), having a partner who is always travelling [28] (*n* = 1), and having a partner who did not test [28] (*n* = 1) were all reported as a barrier to returning for results. Domestic violence (abused by a partner) [28] and rape [16, 29] were reported as barriers to a return for test results in three studies.

The association between a return for results and the availability of a social network has been studied in six studies. On the one hand, it appears that having one or more gay friends [37], having a counsellor [40], or knowing someone infected with HIV [35] are barriers to a return for results. On the other hand, having social support [40] (friends) and lacking a family confidant [38] were reported as facilitators of a return for test results.

### 3.7. Contextual Level

A negative association was found between the return for HIV test results and having a confidential test in one study [12]. The same negative association was found when the testing was done in facilities such as family planning clinics [27, 36] (*n* = 2), a detention facility [27] (*n* = 1), a primary care clinic [27] (*n* = 1), an HIV testing clinic [27] (*n* = 1), a mobile clinic [27] (*n* = 1), a prenatal/obstetric clinic [36] (*n* = 1), a drug treatment center [36] (*n* = 1), a health department [27, 36] (*n* = 2), an outpatient medical service [16] (*n* = 1), and a sexual health clinic [20] (*n* = 1). However, the association was positive in the case of a physician clinic [36] (*n* = 1) and a college [36] (*n* = 1). Other organizational factors, such as the year of testing [22] (*n* = 1), condom distribution during the visit [46] (*n* = 1), or having tested in a center located in the same city as the treatment center [46] (*n* = 1) and not having pretest counselling, emerged as facilitators of a return for results.

### 3.8. Combined Effect Sizes of Factors Associated with a Return for HIV Test Results

The pool estimates and sensitivity analysis of the association of the return for HIV test results with certain factors, including gender (men versus women) and race (black versus white) for studies conducted in the USA, injection drug use (no versus yes), HIV test results (positive versus negative), and HIV testing history (no versus yes), are shown in Table 3. The combined analysis showed that being female [8, 18–20, 22, 30, 34, 35] is significantly associated with a return for results (OR = 0.86, 95% CI = 0.77–0.96) when studies with specific population (MSM, HIV negative, pregnant women) are excluded. In the studies from the USA, black people tend to return less frequently for their results than white people (OR = 0.76, 95% CI = 0.64–0.90) [12, 18, 22, 27, 34–36, 38, 39]. There is no significant association between returning for test results and HIV test results or HIV testing history. Finally, the association between injection drug use and returning for test results was significant (OR = 0.85, 95% CI = 0.75–0.96) [8, 12, 14, 22, 41] when only the general population was considered. Thus, being an IUD appears as a barrier for returning for HIV test results.

## 4. Discussion 

The objective of this review was to report the factors that were statistically associated with the return for HIV test results in different studies, regardless of the target population, the HIV test method used (standard or rapid tests), the waiting time for results, or the country. Despite these different contexts, periods, and populations, the majority of studies considered the same factors. The vast majority of reported factors are found at the individual level (sociodemographic characteristics, risk behaviours, individual risk perception, and test results). Very few studies have reported contextual factors, such as organizational factors, policies, economic factors, or social factors.

The differences in statistical analysis (classification, categorization, and reference group) introduced a great deal of heterogeneity with respect to the studies. Thus, it was not possible to combine effect sizes for all factors. The factors not included in the meta-analysis were grouped into barriers and facilitators based on their statistical association with the dependent variable (return for HIV test results).

Although the factors have been grouped into categories according to the ecological model, it is important to specify, in accordance with the socioecological approach, that the categories are not exclusive but rather influence each other.

### 4.1. Sociodemographic Factors

Age and level of education acted both as barriers and facilitators. However, the trend indicates firstly that young people and individuals with a low level of education were less likely to return for their results. Indeed, there is evidence that young people are often less informed about HIV and also exhibited a lower rate of HIV testing than adults [45, 46, 52]. They are unaware of their risk behaviours and are less likely to return for their test results. Conversely, individuals with higher levels of education can better understand the importance of screening [44, 53] and are more likely to return for their results.

### 4.2. Risk Behaviours

The literature has shown an association between the return for test results and risk behaviours [52, 54]. In fact, people who display risk behaviours can also develop fear and anxiety with respect to knowing their test results. In these circumstances, they are less likely to return for their results even if they had the courage to get tested. Thus, in this review, the positive test result, injection drug use, a high number of sexual partners, getting paid to have sex, and having symptoms of sexually transmitted infections (STIs) at a testing visit were reported as barriers to returning for test results.

### 4.3. Perceived Risk

Studies that have examined the association between perceived risk and a return for test results are sometimes contradictory. Indeed, some studies have shown that people who have a high-perceived risk of contracting HIV were more likely to return for their results [33, 55]. Other studies have shown that people with a low perceived risk do not return for their results [10, 14, 26, 27]. This second situation might be explained by the fact that many people at high risk of contracting HIV do not perceive themselves as at risk [56–58]. Therefore, they do not see the importance of returning for their results and knowing their status. This is why it is recommended that the education of individuals be intensified in order to foster a high and precise perception of risk.

### 4.4. Interpersonal Factors

The sexual partner's risk behaviours were the most frequent group of factors influencing a return for test results. Furthermore, having social support, having an HIV infected partner, or being a client of a sex worker have been reported as factors that encourage people to return for their results. In fact, having sex with a high-risk person might increase the perceived risk, which leads the exposed person to learn his or her HIV status [59]. On the other hand, the family and social network provide social support and reinforce social norms [60] that might encourage a return for results. In contrast, being a member of a social network of people at risk, such as a partner of a sex worker, of an alcoholic, or of a drug user, having gay friends, or knowing someone infected by HIV tend to hinder a return for results. These risk groups often experience discrimination and stigmatization [1, 61]. Therefore, they are less likely to get tested, to return for their results, to disclose their HIV status to others, to adopt preventive behaviours, or to access treatment services, care, and support. Finally, domestic violence (intimate partner violence) and sexual assault also hinder a return for results. Despite the implementation of strategies that enable women to get tested at opportune moments such as during pregnancy or childbirth, domestic violence remains a barrier for the entire testing process [62–64]. First, the female victim of sexual violence is afraid to return for her results and know her HIV status because she is afraid of being rejected by her partner who can blame her for having tested without his consent and for being responsible for his contamination in the case of a positive result [62, 64, 65]. Second, the feeling of guilt and fear of victimization and stigmatization experienced by a raped woman can hinder her return for results even if she could be tested [62, 66, 67].

### 4.5. Contextual Factors

The HIV testing center and its characteristics were the most frequent contextual factors reported in different studies. Getting tested in most of the sites appeared as a barrier to returning for test results. One reason may be the type of screening test offered at these sites. Many of the studies in this review were conducted before the use of rapid tests. Recently, several HIV testing centers in developed countries and in developing countries have reported an increase in the demand for testing, the proportion of people who received posttest counselling, and the knowledge of status following the introduction of rapid tests [68–72]. Other studies also showed that clients prefer the centers where they can receive their results without delay on the same day [73–75]. However, it is also reported that when the testing center is linked with the treatment, the pretest counselling is done well, and there is distribution of condoms, this set of factors encourages people to return for their results [1, 7, 32, 53].

This literature review has some limitations. Firstly, the differences in the measurement of factors and the specificity of certain populations (injection drug users, pregnant women, female sex workers, and men who have sex with men) introduce heterogeneity and do not allow meta-analyses for all factors. Secondly, most of the studies were conducted before the advent of rapid testing, but nowadays HIV testing is performed by rapid tests. Therefore, the issue of failure to return for HIV test results is only important in very specific contexts. The use of rapid tests might change the distribution and frequency of certain factors.

A majority of the studies were conducted in the USA The countries of Sub-Saharan Africa, which represent 2/3 of infected people worldwide, do not often publish their results, or only publish their results in local journal articles, which are not indexed in most databases.

Our search strategy was limited to publications in English and French. Only articles published in peer-reviewed journals were considered; grey literature and conference proceedings were not. This may have some implications for the external validity of our results. However, the review included a large number of studies, covering different regions, a broad range of populations, and barriers and facilitators with respect to returning for HIV test results. Furthermore, to the best of our knowledge, this is the first review to focus on factors associated with returning for HIV test results.

## 5. Conclusion

Helping more people learn their HIV status requires the strengthening of counselling and testing services. Returning for HIV test results is the gateway for knowledge and acceptance of HIV status. Various recently implemented strategies, such as provider-initiated testing and counselling, community-based testing and counselling, home-based testing and counselling, and the use of rapid tests, might not be effective if the people tested are not well advised and do not accept their results.

This review identified important factors that need to be addressed to ensure that people return for their HIV test results. Most barriers and facilitators identified were found at the individual level. These results highlight the fundamental role of counselling. Individuals most likely to fail to return for their results must be identified and targeted by the counsellor and delivered a specific message.

## Supplementary Material

The supplementary file presents the number factors associated with failure to return (FTR) and the return for HIV test results per studies. The factors are classified in barriers and facilitators.

## Figures and Tables

**Table 1:** Characteristics of the studies.

Reference (short)	Country	Aim	Population	Outcomes delay	Sample (*n*)	Study design	FTR/return rate	Factors significantly associated with FTR/return
Slutsker et al. (1992) [12]	USA	Assess the extent of and characteristics associated with FTR for posttest counseling in individuals seeking HIV	General population	30 days	9,644	Cross-sectional	24% failed to return for their test results	Being tested at non-HIV clinic Being aged 10–29 years Blacks Recent IVDU Individuals choosing confidential testing

Wimonsate et al. (2011) [13]	Thailand	Evaluate factors associated with HIV testing history and returning for HIV test results	Men who have sex with men (MSM)	7 days for a maximum of 3 months	2,409	Cross-sectional	24.9% returned to receive the test results	Being classified as MSW Older age (≥29) Lacking a family confidant Self-reported history of STI Testing HIV-negative

Bergenstrom et al. (2007) [14]	Vietnam	Assess factors associated with return to posttest counseling	Injecting drug users (IDUs)	Not mentioned	309	Cross-sectional	54% returned to receive the test results	Residence in Bac Ninh town centre (urban district)

Catania et al. (1990) [15]	USA	To examine social, demographic, and psychological predictors of people who fail to return for their test results	General population	Not mentioned	1,007	Cross-sectional	28% failed to return for their results	HIV test knowledge AIDS anxiety Education Age at the moment of blood transfusion

Desai and Rosenheck (2004) [16]	USA	To determine the rates and predictors of HIV testing and receipt of results among homeless adults with serious mental illness in the initial 3-month period after contact with a community-based case management program	Homeless persons with serious mental illness	3 months	2,135	Longitudinal/cohort	Among those tested, 88.8% reported receiving their test results	Positive association with: Level of education Negative association with: Being disabled Outpatient medical service utilization Having a sexually transmitted disease other than HIV Drug problems at baseline Worsening drug problems over the course of follow-up Frequency of HIV testing during follow-up Prior testing history

Dinh et al. (2005) [17]	Vietnam	To identify the factors associated with declining HIV testing and the failure to return for results	Pregnant women	Not mentioned	266	Cross-sectional	55.3% returned for their results	Educational level below the 12th grade

Ellen et al. (2004) [18]	USA	To determine the posttest counselling (PTC) rates for HIV-infected and uninfected individuals receiving HIV counselling and testing on a mobile STD/HIV screening clinic and to determine whether individuals at highest risk for transmitting their infection were less likely to receive PTC than those at lower risk for transmitting	HIV-positive individuals HIV-negative individuals	14 days	2,022	Cross-sectional	66% (infected), 46% (not infected) returned for their results	Among not infected: (i) Being female (ii) Drug treatment in last 3 months (iii) Engaged in sex work over 3 months ago (iv) Having engaged in sex work in last 3 months Among infected: (i) Drug treatment in last 3 months

Erbelding et al. (2004) [19]	USA	To analyse data on STD clinic patients undergoing HIV testing between 1994 ± 1998 who tested HIV-negative to describe characteristics associated with “nonreturn” for results	HIV-negative individuals	1 week	31,777	Retrospective cross-sectional	48% returned for their results	Age < 30 Reason for initial test visit (HIV testing, STD symptoms, contact to STD, STD test positive, check-up) Risk behaviours (ever had same sex contact, ever used injection drugs, ever used inhaled cocaine, ever exchanged sex for money/drugs, ever had sex partner who used injection drugs, ever had sex partner with HIV/AIDS, ever had partner who exchanged sex for money/drugs, ≥ two partners, past month) STD at test visit (gonorrhea, syphilis, other STD)

Healey et al. (2010) [20]	Australia	To assess the proportion of patients who returned for HIV results and factors predicting return	General population	Within 4 weeks	159	Retrospective cross-sectional	45% returned for their results	Male gender Attending the men-only outreach clinic Having a first HIV test at the clinic Having sex overseas in the past year

Hightow et al. (2003) [21]	USA	To assess the prevalence and predictors of receiving HIV test results	General population	2 weeks later	508	Retrospective cohort	55% (overall) failed to return for test result	HIV testing history STD diagnosis (HPV) Demographic characteristics (black race)

Hong et al. (2011) [8]	Vietnam	To assess whether this program was reaching its targeted populations and examined factors that influenced their service utilization	General population	1 week	158,888	Retrospective cross-sectional	3.5% indicated failure to return for test results	Clients from the Central Highlands provinces Those who were referred by peer educators Those reporting no receipt of prior test results

Kinsler et al. (2007) [22]	USA	To examine time trends of FTR for HIV test results among a mobile van population in Los Angeles	General population	7 days	7,724	Retrospective cross-sectional	FTR by years were as follows: 18% (1997); 24% (1998); 28% (1999); 37% (2000); 43% (2001); 37% (2002); 41% (2003); 35% (2004)	Those testing positive Women Black Latino Those older than 20 years of age

Laanani et al. (2015) [10]	France	To assess factors associated with FTR for HIV test results in a free and anonymous screening centre (CDAG) in Paris	General population	3 days after the blood sampling	710	Cross-sectional	6.5% (overall) failed to return	People who did not specify their birthplace People who were living outside of the Paris region Having sex with 6 partners or more during the last year Reporting visiting for clinical symptoms Having absolutely no self-perceived risk Having a higher self-perceived risk

Ladner et al. (1996) [23]	Rwanda	To identify factors associated with failure to return for HIV posttest To assess the prevalence and predictors of counseling in pregnant women in Kigali	Pregnant women	Approximately 2 weeks later	765	Longitudinal/cohort	Among 68.8% returned for their results	Positive HIV test result

Lazebnik et al. (2001) [24]	USA	To quantify the proportion of adolescents who return for their test results and posttest counseling in a free clinic setting and to identify the characteristics predicting their return	Adolescents	Within 10 days	285	Retrospective cohort	42% (overall) returned for their results	Having unprotected sex while using drugs or alcohol Coming to clinic only for HIV testing Having private health insurance

Machekano et al. (2000) [25]	Zimbabwe	To describe the correlates of HIV test results-seeking behavior and the use of partner counseling testing services among study participants	Male factory workers	After 2 weeks	3,383	Longitudinal/cohort	56% returned for results	Reporting an STD Lower monthly salary

Mmbaga et al. (2009) [26]	Tanzania	To assess the prevalence and predictors of failure to return for HIV posttest counseling among adults	General population	2 weeks after blood sample collection	890 (women) 601 (men) Total = 1,491	Cross-sectional	50.9% failed to return for results	Lack of formal education or no education Lack of HIV/AIDS transmission knowledge Lack of knowledge of antiretroviral therapy availability Perceived low risk of HIV infection Men who were not ready to share their HIV results with their partners Individuals who reported recent (past month) involvement in multiple sexual partners Failure to use condom during last casual sex among men HIV seropositive individuals

Molitor et al. (1999) [27]	USA	To determine the primary predictors of FTR for each of eight types of publicly funded sites in California Predictors of FTR were examined from among those variables assessed during the pretest, risk assessment session.	General population	2 weeks later	370,220	Retrospective cross-sectional	The FTR rate for the entire sample was 16.4%	The type of site at which testing took place (mobile testing,) Race/ethnicity (african american) Risk behavior (IUD) Having sex for money or drugs Blood transfusion Sex partner at risk Multiple sex partners History of FTR Age (<20)

Msuya et al. (2006) [28]	Tanzania	To determine the predictors of failure to return (FTR) for HIV posttest results among pregnant women	Pregnant women	After 1 week	2,413	Longitudinal/cohort	7% failed to return	Failure to bring the partner Site of recruitment Occasional alcohol consumption Age of 25 to 29 years Gestation age of 29 weeks or longer Alcohol intake by male partner Male who frequently travel Never having discussed reproductive health issues with their partners

Sahlu et al. (1999) [29]	Ethiopia	To describe sexual behaviours, perception of risk of HIV infection, and factors associated with attending HIV posttest counseling (PTC) among Ethiopian adults	Factory workers	30 days later at the project's clinic	407 (male) 344 (female) Total = 751	Longitudinal/cohort	43.5% returned for the test results	Positive association with: Being a manual worker History of recent casual sexual relationships Good knowledge of HIV infection Belief that medical follow-up improves the course of HIV infection History of genital symptoms Positive syphilis serology Recent weight loss Negative association with: Belief that HIV/AIDS can be cured Never having been married Having five or less children Having been raped Having used health facilities in the past year

Sesay and Chien (2012) [30]	Gambia	To describe the proportion of clients failing to return for an HIV-test result and to examine the factors associated with failure to return (FTR)	General population	Following day after testing	1,755	Retrospective cross-sectional	30% (overall) failed to return	Male gender Age under 18 Senegalese and persons of others nationality Participants with primary and secondary school education Having ever used condoms Those who resided in a urban area

Sorin et al. (1996) [31]	USA	Analyses predictors of women's decisions to accept testing voluntarily and return for their test results	Pregnant women	Not mentioned	6,104	Retrospective cross-sectional	50% of those tested returned for posttest counseling	Minorities (Blacks, Hispanics) Self-paying clients/uninsured Women receiving less than five prenatal care visits during their pregnancies Receiving a positive test result

Melo et al. (2012) [32]	Brazil	To examine characteristics associated with rates of psychiatric patients receiving their serologic test results for HIV and other sexually transmitted infections	Psychiatric patients	After a maximum of 4 attempts	2,080	Cross-sectional	79.6% (overall) returned for the results	Living in the same city where the treatment centers were located Being single Not having heard of AIDS Not having been previously HIV tested Regular free distribution of condoms to patients

Stein and Nyamathi (2000) [33]	USA	To assess gender differences in psychosocial and behavioural predictors of HIV testing and returning for results in a high-risk sample	Heterosexual persons at high risk for HIV	Not mentioned	428 (male) 621 (female) Total = 1,049	Cross-sectional	Men: 17% and women: 15% failed to return	Injection drug use Self-esteem Social support AIDS knowledge Poor access Perceived risk Sexual risk Negative coping Positive coping History of HIV test and return for test results

Sullivan et al. (2004) [34]	USA	To document the frequency of self-reported failure to return for HIV test results (FTR) and associated reasons among persons at high risk for HIV infection	MSM, IDUs, high risk heterosexuals (HRHs)	Not mentioned	782 (MSM) 697 (HRHs) 762 (IDUs) Total = 2,241	Cross-sectional	Overall: 18.4% failed to return	Among HRHs: Higher educational attainment (<high school) Full time employment (<35 hours)

Tao et al. (1999) [35]	USA	To determine the frequency and predictors of receipt of HIV test results for all tested persons in 1994 and 1995 in US publicly funded counseling and testing programs	General population	Not mentioned	19,127 (1994) 16,848 (1995) Total = 35,975	Cross-sectional	In 1994, 12.5%(±1.0%) and 13.3% (±0.9%) in 1995 had not received their test result	People whose test was compulsory People for whom test was required for hospitalization or surgery Black people recommended by doctor Health department Sex partner Other reasons

Valdiserri et al. (1993) [36]	USA	To identify factors independently associated with returning for HIV result disclosure and posttest counseling services	General population	Not mentioned	557,967	Retrospective cross-sectional	On average, 63% of persons who received HIV pretest counseling and testing returned to learn their test results and obtain posttest counseling	STD clinic Family planning clinic Tuberculosis clinic Private Physician College education Race or ethnicity (Black) Age 13–19 Age 50 or older Age 40–49 MSM MSM and IDU HIV positive Sex partner at risk Blood recipient

Van De Ven et al. (2000) [37]	Australia	To know the extent of HIV testing overall and the factors associated with not having HIV test results	MSM	Not mentioned	5,299	Cross-sectional	Overall, 13.3% of the men did not have HIV test results	Younger age City of residence (Melbourne and Perth) Occupation (clerical/sales and plant operator/labourer) Bisexual/heterosexual Fewer gay friends Fewer male sexual partners Sex with regular and casual partners (having anal intercourse per se with such partners)

Wiley et al. (1998) [38]	USA	To determine the characteristics associated with not receiving an HIV test result in an STD clinic setting	General population	7 to 10 days later	6,988	Cross-sectional	49% did not receive their results	Requesting an HIV test No tested previously for HIV infection Blacks Hispanic/Latino Others

Xu et al. (2011) [9]	China	To analyse the risk factors associated with previous HIV-testing and current posttest follow-up among FSWs in Kaiyuan and Gejii, Yunnan, China	Female sex workers	4 weeks	1,621	Longitudinal/cohort	Overall: 53.3% failed to return	≥9 years of school <5 clients in the recent week Were from another province Were from another city

Ziek et al. (2000) [39]	USA	To examine demographic and behavioral factors related to taking an HIV test and returning for results in a sample of out-of-treatment IDUs and crack smokers	IDUs and crack smokers	3 weeks	927	Longitudinal/cohort	81% return to receive test results	Age per decade High school graduated Ever exchanged sex for money Ever in prison

Cartoux et al. (1998) [40]	Ivory Coast and Burkina Faso	To evaluate the attitude of pregnant women towards HIV testing in two cities of West Africa: Abidjan, Côte d'Ivoire, and Bobo-Dioulasso, Burkina Faso	Pregnant women	2 or 3 weeks	9,724	Longitudinal/cohort	41.6 % in Abidjan and 18.25% in Bobo-Dioulasso, failed to return	Counselor's attitude Weeks of gestation Muslim Positive HIV infection status Being merchant profession Being employee (other) Being 3–6 years in couple Living in Bobo-Dioulasso Having knowledge of AIDS Not using condom for prevention

Pahlavan et al. (2015) [11]	France	To assess the proportion of FTR for an HIV-positive test result among those who tested positive and to identify risk factors associated with FTR	HIV-positive patients	Patient who did follow up for 1 year	509	Retrospective cross-sectional	FTR rate was 14.5%	Heterosexual orientation

Chan et al. (2007) [41]	Australia	To examine the proportion and characteristics of patients who returned to a large sexual health service to obtain their HIV test results	Populations at high risk of STIs and HIV	Within one month	8,715	Cross-sectional study	Overall 79.7% returned within one month of HIV testing,	For male gender Being an HIV contact MSM Having more than five sex partners or overseas sex partners in the past 12 months being overseas born

Healey et al. (2010) [20]	Australia	To assess the proportion of patients who returned for HIV results and factors predicting return	General population	Within four weeks	Files of 218 patients	Cross-sectional study (a retrospective review of patients' files)	45% returned for their results	Male gender Attending the men-only outreach clinic Having the first HIV test at the clinic Having sex with overseas-born individual in the past year

**(a):** 

Study	Selection				Comparability	Outcome		Total (/10)
	Representativeness	Sample size	Nonrespondents	Ascertaining of exposure		Assessment of outcome	Statistical test	
Bell et al. (1997) [42]	—	—	—	*∗*	*∗*	*∗*	—	3
Bergenstrom et al. (2007) [14]	*∗*	—	—	—	*∗∗*	*∗∗*	*∗*	6
Catania et al. (1990) [15]	—	—	—	*∗*	*∗∗*	*∗∗*	*∗*	6
Chan et al. (2007) [41]	*∗*	*∗*	—	*∗*	*∗*	*∗∗*	*∗*	7
Dinh et al. (2005) [17]	—	—	—	*∗*	*∗∗*	*∗∗*	*∗*	6
Ellen et al. (2004) [18]	*∗*	—	—	*∗*	*∗∗*	*∗∗*	*∗*	7
Erbelding et al. (2004) [19]	*∗*	*∗*	—	*∗*	*∗∗*	*∗∗*	*∗*	8
Healey et al. (2010) [20]	—	*∗*	—	—	*∗∗*	*∗∗*	—	5
Hong et al. (2011) [8]	*∗*	*∗*	—	*∗*	*∗∗*	*∗∗*	*∗*	8
Kawichai et al. (2006) [43]	*∗*	*∗*	—	*∗*	*∗∗*	*∗∗*	*∗*	8
Kinsler et al. (2007) [22]	*∗*	*∗*	—	*∗*	*∗∗*	*∗∗*	*∗*	8
Laanani et al. (2015) [10]	—	*∗*	*∗*	*∗*	*∗∗*	*∗∗*	*∗*	8
Mmbaga et al. (2009) [26]	*∗*	*∗*	—	*∗*	*∗∗*	*∗∗*	*∗*	8
Molitor et al. (1999) [27]	*∗*	*∗*	—	*∗*	*∗∗*	*∗∗*	*∗*	8
Pahlavan et al. (2015) [11]	—	*∗*	—	*∗*	*∗∗*	*∗∗*	*∗*	7
Sesay and Chien (2012) [30]	—	—	*∗*	*∗*	*∗∗*	*∗∗*	*∗*	7
Slutsker et al. (1992) [12]	*∗*	*∗*	—	*∗*	*∗∗*	*∗∗*	*∗*	8
Sorin et al. (1996) [31]	*∗*	*∗*	—	*∗*	*∗∗*	*∗∗*	*∗*	8
Melo et al. (2012) [32]	*∗*	*∗*	*∗*	*∗*	*∗∗*	*∗∗*	*∗*	9
Stein and Nyamathi (2000) [33]	—	—	—	*∗*	*∗∗*	*∗∗*	*∗*	6
Sullivan et al. (2004) [34]	*∗*	*∗*	—	*∗*	*∗∗*	*∗∗*	*∗*	8
Tao et al. (1999) [35]	*∗*	*∗*	—	*∗*	*∗∗*	*∗*	*∗*	7
Valdiserri et al. (1993) [36]	*∗*	*∗*	—	*∗*	*∗∗*	*∗∗*	*∗*	8
Van De Ven et al. (2000) [37]	—	—	—	—	*∗∗*	*∗*	*∗*	4
Wiley et al. (1998) [38]	*∗*		—	*∗*	*∗∗*	*∗∗*	*∗*	7
Wimonsate et al. (2011) [13]	*∗*	*∗*	—	*∗*	*∗∗*	*∗∗*	*∗*	8

**(b):** 

Study	Selection				Comparability	Outcome			Total (/9)
	Representativeness	Selection of nonexposed cohort	Ascertaining of exposure	Presence of outcome		Assessment of outcome	Follow-up	Adequacy of follow-up	
Cartoux et al. (1998) [40]	*∗*	*∗*	*∗*	*∗*	*∗∗*	*∗*	*∗*	*∗*	9
Desai and Rosenheck (2004) [16]	—	—	*∗*	*∗*	*∗∗*	*∗*	*∗*	*∗*	7
Hightow et al. (2003) [21]	*∗*	*∗*	*∗*	*∗*	*∗∗*	*∗*	*∗*	*∗*	9
Ladner et al. (1996) [23]	*∗*	*∗*	*∗*	*∗*	*∗∗*	*∗*	*∗*	*∗*	9
Lazebnik et al. (2001) [24]	—	—	*∗*	*∗*	*∗∗*	*∗*	*∗*	*∗*	7
Machekano et al. (2000) [25]	—	*∗*	*∗*	*∗*	*∗∗*	*∗*	*∗*	*∗*	8
Msuya et al. (2006) [28]	—	—	*∗*	*∗*	*∗∗*	*∗*	*∗*	*∗*	7
Sahlu et al. (1999) [29]	—	—	*∗*	*∗*	*∗∗*	*∗*	*∗*	*∗*	7
Xu et al. (2011) [9]	—	—	*∗*	*∗*	*∗∗*	*∗*	*∗*	*∗*	7
Ziek et al. (2000) [39]	—	—	*∗*	*∗*	*∗∗*	*∗*	*∗*	*∗*	7

**Table 3:** Number of quotes per factor classified by barrier and facilitator with respect to return for HIV test results.

Factors	Barrier	Facilitator	Insignificant	Total
*Individual factors*	*136*	*81*	*111*	*328*

*(A) Sociodemographic characteristics*	*57*	*35*	*61*	*153*
*(1) Age*	*14*	*11*	*19*	*44*
(1.1) Younger age	1	—	—	1
(1.2) Older age	—	1	—	1
(1.3) Childhood 12 years and under	—	2	—	2
(1.4) Under 30 years including 34 years and under	10	1	7	18
(1.5) 30 years old and over	3	7	12	22
*(2) Gender*	*6*	*—*	*3*	*9*
(2.1) Female	3	—	1	4
(2.2) Male	3	—	2	5
*(3) Sexual orientation*	*4*	*1*	*2*	*7*
(3.1) Heterosexual	2	1	1	4
(3.2) Bisexual or heterosexual	1	—	—	1
(3.3) Transgender	—	—	1	1
(3.4) Other orientation	1	—	—	1
*(4) Education*	*5*	*4*	*3*	*12*
(4.1) None or low education	4	—	2	6
(4.2) High school education and more	1	4	1	6
*(5) Marital status*	*1*	*3*	*6*	*10*
(5.1) Single/unmarried	1	1	1	3
(5.2) Married/living with partner	—	1	1	2
(5.3) Divorced/separated	—	—	3	3
(5.4) Widowed	—	1	1	2
*(6) Occupation*	*1*	*3*	*8*	*12*
(6.1) Working	—	3	8	11
(6.2) Other occupation	1	—	—	1
*(7) Living condition*	*1*	*—*	*1*	*2*
(7.1) Incarcerated	1	—	—	1
(7.2) Homeless	—	—	1	1
*(8) Residence*	*2*	*4*	*5*	*11*
(8.1) Urban	1	4	5	10
(8.2) Rural	1	—	—	1
*(9) Place of birth*	*3*	*7*	*2*	*12*
(9.1) From other city	—	1	—	1
(9.2) From other province	—	1	—	1
(9.3) Abroad/overseas	—	1	1	2
(9.4) Different parts of Vietnam	3	3	1	7
(9.5) Other place of birth	—	1	—	1
*(10) Nationality*	*2*	*—*	*1*	*3*
(10.1) From another country	2	—	—	2
(10.2) From a tribe	—	—	1	1
*(11) Ethnicity*	*13*	*2*	*8*	*23*
(11.1) Black	5	1	3	9
(11.2) Hispanic/Latino	5	—	2	7
(11.3) Asian/Pacific islander	—	1	2	3
(11.4) Native American/Alaskan	2	—	1	3
(11.5) White	1	—	—	1
*(12) Religion*	*1*	*—*	*3*	*4*
(12.1) Christian	—	—	3	3
(12.2) Muslim	1	—	—	1
*(13) Weeks of gestation*	*3*	*—*	*—*	*3*
(13.1) Fewer than 35	1	—	—	1
(13.2) More than 35	2	—	—	2
*(14) Having 5 or fewer children*	*1*	*—*	*—*	*1*

*(B) Risky behaviors*	*39*	*21*	*24*	*84*
(I) Self-reported risks	35	20	24	79
*(1) Number of sex partners*	*7*	*2*	*8*	*17*
(1.1) One	—	—	2	2
(1.2) 2 to 5	1	1	2	4
(1.3) More than 5	3	1	4	8
(1.4) Multiple	3	—	—	3
*(2) MSM*	*1*	*2*	*3*	*6*
*(3) Sex work*	*5*	*3*	*1*	*9*
*(4) Condom use*	*2*	*3*	*4*	*9*
(4.1) Protected sex	1	2	3	6
(4.2) Unprotected sex	1	1	1	3
*(5) Particular sex behavior*	*1*	*2*	*—*	*3*
(5.1) Oral sex	1	—	—	1
(5.2) Overseas partners	—	1	—	1
(5.3) Sex with casual partner	—	1	—	1
*(6) Ever had sex*	*—*	*1*	*1*	*2*
(6.1) Had sex	—	—	1	1
(6.2) No sex	—	1	—	1
*(7) History of STD other than HIV*	*5*	*4*	*1*	*10*
*(8) IDU*	*8*	*1*	*3*	*12*
(8.1) User	8	1	2	11
(8.2) Nonuser	—	—	1	1
*(9) Other drug use*	*3*	*—*	*1*	*4*
*(10) Blood-related risks*	*2*	*1*	*1*	*4*
(10.1) Blood transfusion	1	1	—	2
(10.2) Blood contact through behavior	1	—	—	1
(10.3) Hemophilia	—	—	1	1
*(11) Alcohol consumption*	*1*	*—*	*1*	*2*
(11.1) Occasional drinker	1	—	—	1
(11.2) Daily drinker	—	—	1	1
*(12) Other self-reported risks*	*—*	*1*	*—*	*1*
(II) Symptoms	4	1	—	5
*(1) Genital/anal symptoms*	*1*	*—*	*—*	*1*
*(2) Loss of weight*	*1*	*—*	*—*	*1*
*(3) Prenatal care*	*1*	*1*	*—*	*2*
(3.1) Low prenatal care	1	—	—	1
(3.2) Had prenatal care	—	1	—	1
*(4) Other symptoms*	*1*	*—*	*—*	*1*

*(C) Perception of risk*	*5*	*1*	*8*	*14*
*(1) Level of perception*	*5*	*1*	*8*	*14*
(1.1) No risk	2	—	—	2
(1.2) Low risk	1	—	2	3
(1.3) Medium risk/some risk	1	1	1	3
(1.4) High risk	1	—	4	5
(1.5) Unknown risk	—	—	1	1

*(D) HIV knowledge*	*6*	*2*	*—*	*8*
*(1) Level of knowledge*	*6*	*2*	*—*	*8*
(1.1) No/low knowledge	3	1	—	4
(1.2) Have knowledge	2	1	—	3
(1.3) No knowledge about ART availability	1	—	—	1

*(E) Visiting reason*	*8*	*10*	*6*	*24*
*(1) Compulsory*	*—*	*1*	*—*	*1*
*(2) HIV testing*	*2*	*3*	*—*	*5*
*(3) STI screening*	*—*	*1*	*—*	*1*
*(4) Have symptoms*	*1*	*—*	*—*	*1*
*(5) Risk behavior taking*	*1*	*1*	*1*	*3*
*(6) Partner-related reasons*	*1*	*1*	*1*	*3*
(6.1) Partner is infected	—	—	1	1
(6.2) Partner is at high risk	—	1	—	1
(6.3) Current relationship	1	—	—	1
*(7) Casual contact with HIV-infected person*	*—*	*—*	*1*	*1*
*(8) Recommended by professional*	*1*	*1*	*1*	*3*
*(9) Clinical procedure*	*—*	*1*	*—*	*1*
*(10) Blood transfusion*	*1*	*—*	*—*	*1*
*(11) Unknown/other*	*1*	*1*	*2*	*4*

*(F) HIV test result*	*4*	*5*	*4*	*13*
*(1) Positive HIV test*	*4*	*4*	*2*	*10*
*(2) Negative HIV test*	*—*	*1*	*2*	*3*

*(G) Testing history*	*8*	*2*	*6*	*16*
*(1) Prior HIV testing*	*4*	*2*	*3*	*9*
(1.1) Tested previously	2	1	3	6
(1.2) Not tested previously	2	1	—	3
*(2) Prior HIV testing status*	*1*	*—*	*1*	*2*
(2.1) Prior negative HIV test	1	—	—	1
(2.2) Prior negative HIV test	—	—	1	1
*(3) Previously FTR/return*	*3*	*—*	*2*	*5*
(3.1) Previously FTR	3	—	1	4
(3.2) Previously return	—	—	1	1

*(H) Psychosocial factors*	*7*	*2*	*—*	*9*
*(1) Beliefs*	*3*	*—*	*—*	*3*
(1.1) Did not believe in self-prevention from HIV	1	—	—	1
(1.2) Belief that HIV can be cured	1	—	—	1
(1.3) Belief that medical follow-up can improve course of HIV	1	—	—	1
*(2) Psychological characteristics*	*4*	*2*	*—*	*6*
(2.1) Self-esteem	—	1	—	1
(2.2) Positive coping skills	—	1	—	1
(2.3) Anxiety about HIV	4	—	—	4

*(I) Others individual factors*	*2*	*3*	*2*	*7*
*(1) Other age-related factors*	*—*	*—*	*2*	*2*
(1.1) Over 17 at outset of sexual activity	—	—	1	1
(1.2) Over 17 at marriage/cohabitation	—	—	1	1
*(2) Being disabled*	*1*	*—*	*—*	*1*
*(3) Treated for drugs*	*—*	*2*	*—*	*2*
*(4) Health coverage*	*1*	*1*	*—*	*2*
(4.1) Private coverage	—	1	—	1
(4.2) No coverage	1	—	—	1

*Interpersonal factors*	*19*	*12*	*15*	*46*

*(A) Risky partner behaviors*	*9*	*6*	*7*	*22*
*(1) Partner STD infections*	*1*	*3*	*1*	*5*
(1.1) HIV-infected	—	3	1	4
(1.2) STD-infected	1	—	—	1
*(2) Partner alcohol/drug use*	*2*	*—*	*2*	*4*
(2.1) IDU	1	—	2	3
(2.2) Alcohol consumer	1	—	—	1
*(3) Partner sexuality*	*—*	*—*	*2*	*2*
(3.1) MSM	—	—	1	1
(3.2) Bisexuality	—	—	1	1
*(4) Partner and sex work*	*2*	*1*	*1*	*4*
(4.1) Sex worker	2	—	1	3
(4.2) Client of sex work	—	1	—	1
*(5) Partner has multiple sex partners*	*—*	*—*	*1*	*1*
*(6) Partner is traveling*	*1*	*—*	*—*	*1*
*(7) Partner did not test*	*1*	*—*	*—*	*1*
*(8) Partner has other risks/unknown risks*	*2*	*2*	*—*	*4*

*(B) Social support*	*4*	*5*	*2*	*11*
*(1) Family relationship*	*—*	*2*	*1*	*3*
(1.1) Living with nonrelatives	—	—	1	1
(1.2) Living with spouse	—	1	—	1
(1.3) Living with relatives	—	1	—	1
*(2) Number of gay friends*	*3*	*—*	*—*	*3*
(2.1) Few gay friends	1	—	—	1
(2.2) Some gay friends	1	—	—	1
(2.3) Mostly gay friends	1	—	—	1
*(3) Having social support*	*—*	*2*	*1*	*3*
*(4) Having a counselor*	*1*	*—*	*—*	*1*
*(5) Lacking a family confidant*	*—*	*1*	*—*	*1*

*(C) Knowledge of person with HIV*	*1*	*—*	*1*	*2*
*(1) Have knowledge of someone with HIV*	*1*	*—*	*1*	*2*

*(D) Other interpersonal factors*	*5*	*1*	*5*	*11*
*(1) Partner age*	*—*	*—*	*3*	*3*
(1.1) 25 to 34 years old	—	—	1	1
(1.2) 34 to 71 years old	—	—	1	1
(1.3) Unknown	—	—	1	1
*(2) Years in couple*	*1*	*—*	*1*	*2*
(2.1) 3 to 6 years	1	—	—	1
(2.2) 7 years and more	—	—	1	1
*(3) Communication*	*1*	*1*	*—*	*2*
(3.1) No discussion about reproductive health issues with partner	1	—	—	1
(3.2) Desire to share results	—	1	—	1
*(4) Domestic violence*	*3*	*—*	*1*	*4*
(4.1) Abuse by partner	1	—	—	1
(4.2) Rape	2	—	1	3

*Contextual factors*	*26*	*6*	*1*	*33*

*(1) Type of clinic attended*	*17*	*3*	*1*	*21*
(1.1) Family planning clinic	2	—	—	2
(1.2) STD clinic	2	1	—	3
(1.3) Detention facility	1	—	1	2
(1.4) Primary care clinic	1	—	—	1
(1.5) HIV test clinic	1	—	—	1
(1.6) Mobile clinic	1	—	—	1
(1.7) Prenatal/obstetric clinic	1	—	—	1
(1.8) Drug treatment center	1	—	—	1
(1.9) Health department	2	—	—	2
(1.10) Outpatient medical service	1	—	—	1
(1.11) Private physician	—	1	—	1
(1.12) College	—	1	—	1
(1.13) Base clinic	1	—	—	1
(1.14) Other type of clinic	3	—	—	3
*(2) Clinic visit (to a facility)*	*1*	*—*	*—*	*1*
*(3) Counselling (no pretest counselling)*	*—*	*1*	*—*	*1*
*(4) Year tested*	*7*	*—*	*—*	*7*
(4.1) 1998	1	—	—	1
(4.2) 1999	1	—	—	1
(4.3) 2000	1	—	—	1
(4.4) 2001	1	—	—	1
(4.5) 2002	1	—	—	1
(4.6) 2003	1	—	—	1
(4.7) 2004	1	—	—	1
*(5) Other contextual factors*	*1*	*2*	*—*	*3*
(5.1) Condom distribution	—	1	—	1
(5.2) Same city as treatment center	—	1	—	1
(5.3) Confidential testing	1	—	—	1

*Grand total*	*181*	*99*	*127*	*407*

## References

[1] Obermeyer C. M., Osborn M. (2007). The utilization of testing and counseling for HIV: a review of the social and behavioral evidence. *American Journal of Public Health*.

[2] UNAIDS (2001). *The Impact of Voluntary Counselling and Testing—A Global Review of the Benefits and Challenges*.

[3] UNAIDS

[4] Helleringer S., Kohler H.-P., Frimpong J. A., Mkandawire J. (2009). Increasing uptake of HIV testing and counseling among the poorest in sub-saharan countries through home-based service provision. *Journal of Acquired Immune Deficiency Syndromes*.

[5] European Centre for Disease Prevention and Control (ECDC) (2010). *HIV Testing: Increasing Uptake and Effectiveness in the European Union*.

[6] CDC (2014). *CDC Fact Sheet: HIV Testing in the United States*.

[7] OMS (2009). *Vers un Accès Universel: Étendre les Interventions Prioritaires Liées au VIH/SIDA Dans le Secteur de la Santé: Rapport de Situation 2009*.

[8] Hong N. T. T., Wolfe M. I., Dat T. T. (2011). Utilization of HIV voluntary counseling and testing in Vietnam: an evaluation of 5 years of routine program data for national response. *AIDS Education and Prevention*.

[9] Xu J., Brown K., Ding G. (2011). Factors associated with HIV testing history and HIV-test result follow-up among female sex workers in two cities in Yunnan, China. *Sexually Transmitted Diseases*.

[10] Laanani M., Dozol A., Meyer L. (2015). Factors associated with failure to return for HIV test results in a free and anonymous screening centre. *International Journal of STD & AIDS*.

[11] Pahlavan G., Burdet C., Laouénan C. (2015). Predictors of return rate for an HIV-positive result in a French Voluntary Counseling and Testing centre. *International Journal of STD and AIDS*.

[12] Slutsker I., Klockner R., Fleming D. (1992). Factors associated with failure to return for HIV post-test counseling. *AIDS*.

[13] Wimonsate W., Naorat S., Varangrat A. (2011). Factors associated with HIV testing history and returning for HIV test results among men who have sex with men in Thailand. *AIDS and Behavior*.

[14] Bergenstrom A., Go V., Nam L. V. (2007). Return to post-test counselling by out-of-treatment injecting drug users participating in a cross-sectional survey in north Vietnam. *AIDS Care*.

[15] Catania J. A., Kegeles S. M., Coates T. J. (1990). Towards an understanding of risk behavior: an AIDS risk reduction model (ARRM). *Health Education Quarterly*.

[16] Desai M. M., Rosenheck R. A. (2004). HIV testing and receipt of test results among homeless persons with serious mental illness. *The American Journal of Psychiatry*.

[17] Dinh T.-H., Detels R., Nguyen M. A. (2005). Factors associated with declining HIV testing and failure to return for results among pregnant women in Vietnam. *AIDS*.

[18] Ellen J. M., Liang T. S., Jacob C. A., Erbelding E., Christmyer C. (2004). Post-HIV test counselling of clients of a mobile STD/HIV clinic. *International Journal of STD & AIDS*.

[19] Erbelding E. J., Chung S., Zenilman J. M. (2004). Following-up for HIV test results: what limits return in an STD clinic population?. *International Journal of STD and AIDS*.

[20] Healey L. M., O'Connor C. C., Templeton D. J. (2010). HIV result giving. Is it time to change our thinking?. *Sexual Health*.

[21] Hightow L. B., Miller W. C., Leone P. A., Wohl D., Smurzynski M., Kaplan A. H. (2003). Failure to return for HIV posttest counseling in an STD clinic population. *AIDS Education and Prevention*.

[22] Kinsler J. J., Cunningham W. E., Davis C., Wong M. D. (2007). Time trends in failure to return for HIV test results. *Sexually Transmitted Diseases*.

[23] Ladner J., Leroy V., Msellati P. (1996). A cohort study of factors associated with failure to return for HIV post-test counselling in pregnant women: Kigali, Rwanda, 1992-1993. *AIDS*.

[24] Lazebnik R., Hermida T., Szubski R., Dieterich-Colón S., Grey S. F. (2001). The proportion and characteristics of adolescents who return for anonymous HIV test results. *Sexually Transmitted Diseases*.

[25] Machekano R., McFarland W., Hudes E. S., Bassett M. T., Mbizvo M. T., Katzenstein D. (2000). Correlates of HIV test results seeking and utilization of partner counseling services in a cohort of male factory workers in Zimbabwe. *AIDS and Behavior*.

[26] Mmbaga E. J., Leyna G. H., Mnyika K. S., Hussain A., Klepp K.-I. (2009). Prevalence and predictors of failure to return for HIV-1 post-test counseling in the era of antiretroviral therapy in rural Kilimanjaro, Tanzania: challenges and opportunities. *AIDS Care*.

[27] Molitor F., Bell R. A., Truax S. R., Ruiz J. D., Sun R. K. (1999). Predictors of failure to return for HIV test result and counseling by test site type. *AIDS Education and Prevention*.

[28] Msuya S. E., Mbizvo E., Uriyo J., Stray-Pedersen B., Sam N. E., Hussain A. (2006). Predictors of failure to return for HIV test results among pregnant women in Moshi, Tanzania. *Journal of Acquired Immune Deficiency Syndromes*.

[29] Sahlu T., Kassa E., Agonafer T. (1999). Sexual behaviours, perception of risk of HIV infection, and factors associated with attending HIV post-test counselling in Ethiopia. *AIDS*.

[30] Sesay C., Chien L.-Y. (2012). Analysis of factors associated with failure to return for an HIV-test result in The Gambia. *African Journal of AIDS Research*.

[31] Sorin M. D., Tesoriero J. M., LaChance-McCullough M. L. (1996). Correlates of acceptance of HIV testing and post-test counseling in the obstetrical setting. *AIDS Education and Prevention*.

[32] Melo A. P. S., McKinnon K., Wainberg M. L., César C. C., Guimarães M. D. C. (2012). Psychiatric patients' return for HIV/STI test results in mental health centers. *Revista de Saude Publica*.

[33] Stein J. A., Nyamathi A. (2000). Gender differences in behavioural and psychosocial predictors of HIV testing and return for test results in a high-risk population. *AIDS Care - Psychological and Socio-Medical Aspects of AIDS/HIV*.

[34] Sullivan P. S., Lansky A., Drake A. (2004). Failure to return for HIV test results among persons at high risk for HIV infection: results from a multistate interview project. *Journal of Acquired Immune Deficiency Syndromes*.

[35] Tao G. Y., Branson B. M., Kassler W. J., Cohen R. A. (1999). Rates of receiving HIV test results: data from the U.S. National Health Interview Survey for 1994 and 1995. *Journal of Acquired Immune Deficiency Syndromes*.

[36] Valdiserri R. O., Moore M., Gerber A. R., Campbell C. H., Dillon B. A., West G. R. (1993). A study of clients returning for counseling after HIV testing: implications for improving rates of return. *Public Health Reports*.

[37] Van De Ven P., Prestage G., Knox S., Kippax S. (2000). Gay men in Australia who do not have HIV test results. *International Journal of STD and AIDS*.

[38] Wiley D. J., Frerichs R. R., Ford W. L., Simon P. A. (1998). Failure to learn human immunodeficiency virus test results in Los Angeles public sexually transmitted disease clinics. *Sexually Transmitted Diseases*.

[39] Ziek K., Goldstein M. F., Beardsley M., Deren S., Tortu S. (2000). Factors associated with HIV testing and returning for test results in a sample of out-of-treatment drug users. *Journal of Drug Issues*.

[40] Cartoux M., Msellati P., Meda N. (1998). Attitude of pregnant women towards HIV testing in Abidjan, Cote d'Ivoire and Bobo-Dioulasso, Burkina Faso. *AIDS*.

[41] Chan E., McNulty A., Tribe K. (2007). Who returns for HIV screening test results?. *International Journal of STD and AIDS*.

[42] Bell R. A., Molitor F., Flynn N. (1997). On returning for one's HIV test result: demographic, behavioral and psychological predictors. *AIDS*.

[43] Kawichai S., Celentano D. D., Vongchak T. (2006). HIV voluntary counseling and testing and HIV incidence in male injecting drug users in northern Thailand: evidence of an urgent need for HIV prevention. *Journal of Acquired Immune Deficiency Syndromes*.

[44] Moher D., Shamseer L., Clarke M. (2015). Preferred reporting items for systematic review and meta-analysis protocols (PRISMA-P) 2015 statement. *Systematic Reviews*.

[45] Wells G. A., Shea B., O'Connell D. (2008). *The Newcastle-Ottawa Scale (NOS) for Assessing the Quality of Nonrandomised Studies in Meta-Analyses*.

[46] Shamseer L., Moher D., Clarke M. (2015). Preferred reporting items for systematic review and meta-analysis protocols (PRISMA-P) 2015: elaboration and explanation. *British Medical Journal*.

[47] McLeroy K. R., Bibeau D., Steckler A., Glanz K. (1988). An ecological perspective on health promotion programs. *Health Education Quarterly*.

[48] DerSimonian R., Laird N. (1986). Meta-analysis in clinical trials. *Controlled Clinical Trials*.

[49] Borenstein M., Hedges L. V., Higgins J. P. T., Rothstein H. R. (2009). *Introduction to Meta-Analysis*.

[50] Higgins J. P. T., Thompson S. G. (2002). Quantifying heterogeneity in a meta-analysis. *Statistics in Medicine*.

[51] Higgins J. P. T., Thompson S. G., Deeks J. J., Altman D. G. (2003). Measuring inconsistency in meta-analyses. *British Medical Journal*.

[52] MacPhail C. L., Pettifor A., Coates T., Rees H. (2008). ‘You must do the test to know your status’: attitudes to HIV voluntary counseling and testing for adolescents among South African youth and parents. *Health Education and Behavior*.

[53] Ayoola O. D., Victoria G.-O. C., Bamidele O. (2014). Pattern, challenges and correlates of condom use among Nigerians living with HIV infection. *Asian Pacific Journal of Tropical Biomedicine*.

[54] Reynolds G. L., Fisher D. G., Napper L. E., Marsh K. A., Willey C., Brooks R. (2008). Results from a multiple morbidities testing program offering rapid HIV testing bundled with hepatitis and sexually transmitted infection testing. *Public Health Reports*.

[55] Fichtner R. R., Wolitski R. J., Johnson W. D., Rabins C. B., Fishbein M. (1996). Influence of perceived and assessed risk on STD clinic clients' acceptance of HIV testing, return for test results, and HIV serostatus. *Psychology, Health and Medicine*.

[56] Nunn A., Zaller N., Cornwall A. (2011). Low perceived risk and high HIV prevalence among a predominantly African American population participating in Philadelphia's rapid HIV testing program. *AIDS Patient Care and STDs*.

[57] Koh K. C., Yong L. S. (2014). HIV risk perception, sexual behavior, and HIV prevalence among men-who-have-sex-with-men at a community-based voluntary counseling and testing center in Kuala Lumpur, Malaysia. *Interdisciplinary Perspectives on Infectious Diseases*.

[58] Shiferaw Y., Alemu A., Assefa A., Tesfaye B., Gibermedhin E., Amare M. (2014). Perception of risk of HIV and sexual risk behaviors among university students: implication for planning interventions. *BMC Research Notes*.

[59] Stephenson R., White D., Darbes L., Hoff C., Sullivan P. (2015). HIV Testing Behaviors and Perceptions of Risk of HIV Infection Among MSM with Main Partners. *AIDS and Behavior*.

[60] Kigozi N. G., Heunis J. C., Wouters E., Van Den Berg H. S. (2011). Tuberculosis patients' reasons for, and suggestions to address non-uptake of HIV testing: a cross-sectional study in the Free State Province, South Africa. *BMC Health Services Research*.

[61] Hutchinson A. B., Branson B. M., Kim A., Farnham P. G. (2006). A meta-analysis of the effectiveness of alternative HIV counseling and testing methods to increase knowledge of HIV status. *AIDS*.

[62] WHO (2006). *Addressing Violence against Women in HIV Testing and Counselling: A Meeting Report*.

[63] Kiarie J. N., Farquhar C., Richardson B. A. (2006). Domestic violence and prevention of mother-to-child transmission of HIV-1. *AIDS*.

[64] Turan J. M., Bukusi E. A., Onono M., Holzemer W. L., Miller S., Cohen C. R. (2011). HIV/AIDS stigma and refusal of HIV testing among pregnant women in rural Kenya: results from the MAMAS study. *AIDS and Behavior*.

[65] Medley A., Garcia-Moreno C., McGill S., Maman S. (2004). Rates, barriers and outcomes of HIV serostatus disclosure among women in developing countries: implications for prevention of mother-to-child transmission programmes. *Bulletin of the World Health Organization*.

[66] Sayles J. N., Ryan G. W., Silver J. S., Sarkisian C. A., Cunningham W. E. (2007). Experiences of social stigma and implications for healthcare among a diverse population of HIV positive adults. *Journal of Urban Health*.

[67] Beattie T. S. H., Bhattacharjee P., Suresh M., Isac S., Ramesh B. M., Moses S. (2012). Personal, interpersonal and structural challenges to accessing HIV testing, treatment and care services among female sex workers, men who have sex with men and transgenders in Karnataka state, South India.. *Journal of epidemiology and community health*.

[68] Bowles K. E., Clark H. A., Tai E. (2008). Implementing rapid HIV testing in outreach and community settings: results from an advancing HIV prevention demonstration project conducted in seven U.S. cities. *Public Health Reports*.

[69] Fernàndez-Lopez L., Rifà B., Pujol F. (2010). Impact of the introduction of rapid HIV testing in the voluntary counselling and testing sites network of Catalonia, Spain. *International Journal of STD and AIDS*.

[70] Liu A., Kilmarx P. H., Supawitkul S. (2003). Rapid whole-blood finger-stick test for HIV antibody: performance and acceptability among women in Northern Thailand. *Journal of Acquired Immune Deficiency Syndromes*.

[71] Malonza I. M., Richardson B. A., Kreiss J. K., Bwayo J. J., Stewart G. C. J. (2003). The effect of rapid HIV-1 testing on uptake of perinatal HIV-1 interventions: a randomized clinical trial. *AIDS*.

[72] Telles-Dias P. R., Westman S., Fernandez A. E. (2007). Perceptions of HIV rapid testing among injecting drug users in Brazil. *Revista de Saude Publica*.

[73] Kassler W. J., Alwano-Edyegu M. G., Marum E., Biryahwaho B., Kataaha P., Dillon B. (1998). Rapid HIV testing with same-day results: a field trial in Uganda. *International Journal of STD and AIDS*.

[74] Morin S. F., Khumalo-Sakutukwa G., Charlebois E. D. (2006). Removing barriers to knowing HIV status: same-day mobile HIV testing in Zimbabwe. *Journal of Acquired Immune Deficiency Syndromes*.

[75] Spielberg F., Branson B. M., Goldbaum G. M. (2003). Overcoming barriers to HIV testing: preferences for new strategies among clients of a needle exchange, a sexually transmitted disease clinic, and sex venues for men who have sex with men. *Journal of Acquired Immune Deficiency Syndromes*.

