# Supplementary material for: Returning for HIV Test Results: A Systematic Review of Barriers and Facilitators
Source: Int Sch Res Notices. 2016 Dec 15;2016:6304820. doi: 10.1155/2016/6304820 (PMC5198086; doi:10.1155/2016/6304820)
Supplement: Supplementary file 1 — The supplementary file presents the number factors associated with failure to return (FTR) and the return for HIV test results per studies. The factors are classified in barriers and facilitators. [file 6304820.f1.pdf]

Supplementary File 1: Number of factors per study classified by barriers and facilitators with respect to return

Factors	Barrier	Facilitator	Insignificant	Total of factors per study
**Individual factors**				**204**
**A. Socio-demographic characteristics**				**76**
1. Age	7	4	5	16
2. Gender	6	-	3	9
3. Sexual orientation	4	-	2	6
4. Education	4	3	2	8
5. Marital status	1	2	2	5
6. Occupation	1	2	2	5
7. Living condition	1	-	1	1
8. Residence	2	3	1	6
9. Place of birth	1	4	2	4
10. Nationality	1	-	1	2
11. Ethnicity	6	2	5	10
12. Religion	1	-	2	3
13. Weeks of gestation	2	-	-	2
14. Number of children	1	-	-	1
**B. Risky behaviors**	**31**	**19**	**19**	**65**
1. Self-reported risks				
1.1 Number of sex partners	4	2	3	8
1.2 MSM	-	2	3	5
1.3 Sex work	5	3	1	9
1.4 Condom use	2	3	4	8
1.5 Particular sex behavior	1	1	-	2

Factors	Barrier	Facilitator	Insignificant	Total of factors per study
1.6 Ever had sex	-	1	1	2
1.7 History of STD other than HIV	5	4	1	10
1.8 IDU	7	-	3	10
1.9 Other drug use	1	-	1	2
1.10 Blood-related risks	2	1	1	4
1.11 Alcohol consumption	1	-	1	2
1.12 Other self-reported risks	-	1	-	1
2. Symptoms	3	1	-	3
**C. Perception of risk**	**4**	**1**	**4**	**9**
**D. HIV knowledge**	**6**	**2**	**-**	**7**
**E. Visiting reason**	**7**	**5**	**2**	**11**
**F. HIV test result**	**4**	**5**	**4**	**13**
**G. Testing history**	**7**	**2**	**5**	**11**
**H. Psychosocial factors**	**4**	**1**	**-**	**5**
**I. Other individual factors**				
1. Health coverage	1	1	-	2
2. Other individual factors	1	1	1	3
**Interpersonal factors**				**21**
**A. Risky partner behaviors**	**4**	**5**	**2**	**7**
**B. Social support**	**2**	**4**	**2**	**6**
**C. Knowledge of person with HIV**	**-**	**-**	**1**	**1**
**D. Other interpersonal factors**				
1. Partner age	-	-	1	1

Factors	Barrier	Facilitator	Insignificant	Total of factors per study
2. Years in couple	1	-	1	1
3. Communication	1	1	-	2
4. Domestic violence	3	-	1	3
**Contextual factors**				**12**
**1. Type of clinic attended**	**6**	**1**	**1**	**6**
**2. Clinic visit (to a facility)**	**1**	**-**	**-**	**1**
**3. Counselling (no pre-test counselling)**	**-**	**1**	**-**	**1**
**4. Year tested**	**1**	**-**	**-**	**1**
**5. Other contextual factors**				
5.1 Condom distribution	-	1	-	1
5.2 Same city as treatment center	-	1	-	1
5.3 Confidential testing	1	-	-	1
**Grand total**				**237**
